# Effective *in Vitro* Photokilling by Cell-Adhesive Gold Nanorods

**DOI:** 10.3389/fchem.2018.00234

**Published:** 2018-06-22

**Authors:** Álvaro Artiga, Sonia García-Embid, Laura De Matteis, Scott G. Mitchell, Jesús M. de la Fuente

**Affiliations:** ^1^Instituto de Ciencia de Materiales de Aragón, Consejo Superior de Investigaciones Científicas, Universidad de Zaragoza and CIBER-BBN, Zaragoza, Spain; ^2^Instituto de Nanociencia de Aragón, Universidad de Zaragoza and CIBER-BBN, Zaragoza, Spain

**Keywords:** polyoxometalate, gold nanorod, chitosan hydrogel, encapsulation, photothermal therapy, near infra-red

## Abstract

Upon excitation of their localized surface plasmon resonance (LSPR) band, gold nanorods (AuNRs) show a characteristic light-to-heat transduction, a useful and versatile property for a range of biomedical applications such as photothermal therapy, drug delivery, optoacoustic imaging and biosensing, among others. Nanoparticle (NP)-mediated photothermal therapy (PTT) rests on the ability of nanomaterials to convert light energy into heat and can currently be considered as a promising method for selectively destroying tumor cells by (photo)-thermoablation. One inherent limitation to NP-mediated PTT is that the nanoparticles must arrive at the site of action to exert their function and this typically involves cellular internalization. Here we report the use of the Keggin-type polyoxometalate (POM) phosphotungstic acid (PTA) as an inorganic gelling agent for the encapsulation of plasmonic gold nanorods (AuNRs) inside a biocompatible and cell-adhesive chitosan hydrogel matrix. These functional sub-micrometric containers are non-cytotoxic and present the ability to adhere to the cytoplasmic membranes of cells avoiding any need for cellular internalization, rendering them as highly efficient thermoablating agents of eukaryotic cells *in vitro*.

## Introduction

Plasmonic nanoparticles undergo efficient light-to-heat transduction through excitation of their surface plasmon resonance (SPR) band (Abadeer and Murphy, 2016), a characteristic and versatile property that is applied to a range of biomedical applications: for photothermal therapy (Kharlamov et al., 2015), drug delivery (Yin et al., 2015), optoacoustic imaging (Bao et al., 2013), and biosensing (Parolo et al., 2013) among others. The medical field views nanoparticle (NP)-mediated photothermal therapy (PTT) (based on the ability of nanomaterials to convert light energy into heat) as a promising method for selectively destroying tumor cells (thermoablation). Consequently, NP-mediated PTT can be used as a synergic anticancer therapy to weaken tumor cells (when applied in low doses) by making cells more susceptible to other treatments or permitting a reduction in the effective dose of other aggressive therapies such as radio- (Cooper et al., 2014) and chemo-therapies (Dreaden et al., 2011; Liu et al., 2015). Furthermore, PTT is also used as a highly effective tool to improve the applicability of photodynamic therapy (PDT) by reducing side effects and reducing acquired resistance to drugs (Bucharskaya et al., 2016). The local cellular destructive effect of increased temperature corresponds to damage of sub-cellular components, mainly lysosomes, which then release their contents and biomarkers, which initiate the cascade of events that lead to cell death (Pérez-Hernández et al., 2015). Anisotropic gold nanoparticles (AuNPs) are particularly interesting for PTT therapy because they present an efficient absorption of light at near infrared (NIR) wavelengths; while the absorption of biological tissues and cells at these wavelengths is highly decreased (Huang et al., 2009).

One inherent limitation to NP-mediated PTT is that the particles must arrive at the site of action to exert their function and this typically involves internalization inside the cell. For some applications, the functionalization of nanomaterials with biomolecules for targeted therapeutic and diagnostic applications is especially interesting (Conde et al., 2014; Fratila et al., 2014).

Gold nanorods (AuNRs) possess potent hyperthermia properties, however their efficacy is unsatisfactory because of low cellular internalization (Alfranca et al., 2016). Literature clearly shows how post-synthetic functionalization of the particles is almost always required (Fan et al., 2016; Liu et al., 2016). This can in turn result in issues with particle stability, aggregation in cell culture media and so forth; not to mention problems with reproducibility and elevated synthesis costs. In recent years investigators have highlighted the need for reducing the complexity of nanotherapeutics to allow for clinical translation but maintaining their therapeutic effect (Barz, 2015)—a concept which has framed the research presented herein.

Embedding the active photothermal element (in this case the AuNR) in a polymeric cell-adhesive hydrogel would improve the efficacy of the thermal treatment required to destroy cells without the need for cellular internalization. Recent studies have shown how AuNP entrapment in implantable macroscopic hydrogels have shown promising results as efficient photothermal antitumor treatments in mice (Conde et al., 2016).

The naturally occurring polymer chitosan has been selected in this work for the entrapment of AuNR. Chitosan is a mucopolysaccharide obtained by deacetylation of chitin, a polymer extracted from the exoskeletons of crustacean, insects and the cellular wall of fungi. This polymer has low toxicity and immunogenicity and it is biocompatible, biodegradable and bioadhesive (Younes and Rinaudo, 2015). Besides, as chitin is one of the most abundant polymers in nature, chitosan is readily available and cheap. The mucoadhesive properties of chitosan are due to the fact that it is one of the richest natural polymers in amino groups. These groups pose a keen interest in the fabrication of matrices as they are available for their interaction with other molecules (Yi et al., 2005). One of the most important characteristics of chitosan is its capacity to form hydrogels in the presence of salts, which has been widely used in the pharmaceutical industry for drug release, gene therapy or vaccines administration (Nagpal et al., 2010; Younes and Rinaudo, 2015). Chitosan has been used for coating AuNPs for subsequent application in gene delivery (Yang et al., 2015), photothermal ablation (Duan et al., 2014), and non-invasive imaging (Charan et al., 2012). Besides, it has also been employed for the synthesis of nanospheres containing AuNPs and anticancer drugs, to obtain nanocomposites for photothermal-chemotherapies (Chen et al., 2013; Zhang et al., 2016). Since chitosan possesses mucoadhesive properties, there is significant potential in exploring the use of chitosan-based nanomaterials as novel therapies for diseases related with gastrointestinal tract, for example in colorectal cancer (Kang et al., 2017).

In the present work, we have used the ionotropic gelation method for the synthesis of a hybrid organic-inorganic sub-micrometric container based on a chitosan hydrogel. One of the advantages of this method is that it avoids the use of organic solvents; allowing for AuNR entrapment entirely in aqueous solution. The easy and rapid synthesis is based on the interaction of the positively charged amino groups of chitosan with an appropriate anion that acts as an electrostatic crosslinker. In this work polyoxometalates (POMs) were used as anionic gelling agents. POMs represent an extremely diverse group of anionic molecular metal-oxides with promising application to a range of sub-fields of materials science, catalysis, and medicine. They have demonstrated important antibacterial (Bijelic et al., 2018), antiviral and anticancer characteristics (Hasenknopf, 2005; Yamase, 2005). Recently, POM-based chitosan materials have been reported as potent anti-cancer agents (Menon et al., 2011; Shah et al., 2015) and antibacterial materials (Fiorani et al., 2014). Our own research has shown how POMs can act as gelling agents and provide structural stability to hybrid chitosan hydrogels with antibacterial activity (De Matteis et al., 2014). Furthermore, as part of our research we pay particular attention to how nanoparticles interact with cells and tissues in both *in vitro* and *in vivo* environments (Moros et al., 2013). In recent years we have carried out in depth investigations on the photothermal properties of plasmonic nanoparticles for biosensing (Polo et al., 2013), and optoacoustic imaging (Han et al., 2016).

The aim of this work was to use polyoxometalates (POMs) as inorganic gelation agents for the encapsulation of plasmonic nanoparticles (NPs) inside a muchoadhesive hydrogel for optical hyperthermia applications. Here we report the synthesis of a hybrid organic-inorganic sub-micrometric container of phosphotungstic acid (PTA) and gold nanorods (AuNRs) inside a chitosan hydrogel matrix. The resulting functional container is a highly efficient heat mediator that has been used for the photothermoablation of eukaryotic cells *in vitro*.

## Materials and methods

### Materials

Silver nitrate (AgNO_3_) was purchased from Panreac® (Barcelona, Spain). Chitosan (medium molecular weight), hydrogen tetrachloroaurate (III) hydrate, phosphotungstic acid (PTA), sodium borohydride, sodium hydroxide, and hexadecyltrimethylammonium bromide (CTAB) were purchased from Sigma-Aldrich (MI, USA). Complete Dulbeccos's modified Eagle's medium (DMEM), phosphate buffered saline (PBS) and Dulbecco's Phosphate Buffered Saline (DPBS) supplemented with Ca^2+^ and Mg^2+^ were purchased from Lonza® (Basel, Switzerland). Hydroquinone was purchased from Alfa Aesar® (MA, USA). DMEM was supplemented with 2 mM glutamine, 100 U/mL penicillin/streptomycin, and 10% fetal bovine serum (FBS) for their use in cell culture. LIVE/DEAD® Viability/Cytotoxicity Kit was purchased from Invitrogen^TM^ (CA, USA). For the cell viability assays, MTT (3-(4,5-dimethylthiazol-2-yl)-2,5-diphenyltetrazolium bromide) was purchased from Invitrogen^TM^ (CA, USA). Nanorod suspensions were sterilized with 0.22 μm Low Protein Binding Durapore® (PVDF) MilliPore® (MA, USA) filters prior addition to cell cultures and prior chitosan entrapment. Prior to use, all glassware was cleaned with *aqua regia* and washed thoroughly with Milli-Q water from Millipore Q-POD® system.

UV-Vis spectra were acquired employing a Cary 50 Probe® spectrophotometer from Varian (TO, Italy). The composition of the hydrogel and its components was analyzed by Fourier Transform Infrared Spectroscopy analysis in a JASCO FT/IR-4100 Fourier transform infrared spectrometer (Madrid, Spain) in a frequency range of 600–4,000 cm^−1^ with a resolution of 2 cm^−1^ and a scanning number of 32. SEM images were collected using a field emission SEM Inspect F50 with an EDX system INCA PentaFETx3 (FEI Company, Eindhoven, The Netherlands) in an energy range between 10 and 15 keV. Nanomaterials and cells were irradiated using a 3 W Laser Quantum Ventus laser (1,064 nm) (Cheshire, UK) operating at a power output of 1,100 mW, which corresponds with a power per surface of *ca*. 3.3 W cm^−2^ at the sample position. Both bright-field and fluorescence images of the cells were acquired with a Nikon Eclipse Ti (Tokyo, Japan) with PFS system equipped with a phase contrast system and 472 ± 30/520 ± 35 nm (GFP) and 531 ± 46/593 ± 40 nm (TRITC) cube filter connected to NIS-Elements Microscope Imaging Software. The temperature reached by the nanomaterials suspensions under near infrared (NIR) irradiation was monitored using a software program developed by The University of Zaragoza using a Fiber Optic Temperature Sensor TPT-62 (Fiso Technologies Inc., Quebec, Canada). Optical absorbance of the samples at 96-well plate was recorded using a plate reader [ELx800TM, Biotek (Thermo Scientific Multiskan GO UV/Vis microplate spectrophotometer), VT, USA]. For elemental analysis, samples were evaluated by ICP-AES using an Optima 8300 (Perkin Elmer®). Samples were freeze-dried in a Telstar cryodos freeze-dryer (Spain) with an Agilent technologies DS 102 vacuum pump. Dynamic Light Scattering and Electrophoretic mobility (Zeta Potential) of the hybrid matrixes were evaluated using a Brookhaven 90Plus DLS instrument (NY, USA) at a concentration of 0.05 mg/mL in water or KCl 1 mM, respectively.

### Gold nanorod (AuNR) entrapment on chitosan hydrogel (AuNR@CS)

Polyethylene glycol-stabilized gold nanorods (AuNR) were synthesized according to previously reported literature procedure (Alfranca et al., 2016), which in turn was adapted from a synthetic route first reported by Zubarev and co-workers (Vigderman and Zubarev, 2013). For the AuNR entrapment in chitosan hydrogel, 1 mL of a 2 mg/mL (measured by dry weight) aqueous AuNR solution was transferred to a glass vial. Under ultrasonication, 0.5 μmol of PTA (144 μL of a 10 mg/mL) was slowly pipetted into the AuNR solution. 2 mL of 5 mg/mL chitosan in 1% v/v acetic acid was then pipetted into the PTA/AuNR suspension and mixed for 2 min under sonication. This mixture was then added to a separate tube containing 5 mL 50 mM Na_2_SO_4_ solution, under sonication for 2 min. The reaction suspension was centrifuged at 6,700 g for 3 min. The supernatant was discarded and the product was resuspended and centrifuged once more at 4,300 g for 3 min. The precipitate was resuspended in water. All the experiments for AuNR entrapment were performed under sterile conditions in a laminar flow hood (Telstar PV-30/70). The concentration of the sub-micrometric containers in water suspension was obtained by measuring the weight of 0.5 mL of sample after freeze-drying.

### Elemental analysis by inductively coupled plasma atomic emission spectroscopy (ICP-AES)

The gold content of the AuNR@CS hydrogel and AuNR starting material was quantified by inductively coupled plasma atomic emission spectroscopy (ICP-AES). One hundred microliters of the samples were previously digested by addition of 100 μL of a 3:1 sulfuric acid (96%)/hydrogen peroxide (33%) solution and incubated 15 min at room temperature. After that, 300 μL of a 1:3 nitric acid (65%)/hydrochloric acid (37%) solution were added. Samples were incubated for 2 h at room temperature and at 60°C for 15 min and they were finally diluted with Milli-Q water up to 20 mL. All samples were prepared and analyzed in duplicate.

### Fixation for scanning electron microscopy imaging of AuNRs entrapped in chitosan hydrogel (AuNR@CS)

AuNR@CS samples were covalently crosslinked with glutaraldehyde prior to electron microscopy imaging to fix their supramolecular structure. One milligram of the AuNR@CS hydrogel was incubated for 1 h in a 1.5% glutaraldehyde solution in PBS. The nanomaterials were then washed with Milli-Q water *via* three successive centrifugation cycles (5,500 g for 5 min per cycle).

### Heating profiles of AuNRs and AuNR@CS

The heating curves were measured using a homogeneous laser beam adjusted to the bottom area of one single well on each irradiation (96-well-plates). All samples were irradiated at 1,100 mW for 10 min registering the temperature every 5 s and measurements were performed in triplicate. To avoid pre-heating adjacent samples by heat diffusion and/or diffraction of the beam, one empty well was located between sample rows. The temperature probe was rinsed and cooled in Milli-Q water before collecting new data.

Each concentrated stock of nanomaterials was diluted in Milli-Q water to a final volume of 200 μL per well, giving a final concentration of 0, 5, 15, 30, and 60 μg of gold/mL of AuNRs or AuNR@CS. These heating curves were also used to calculate the heating potency and efficiency of both nanomaterials at the studied concentrations. For this purpose, a linear regression of the heating curves during the first 60 s of irradiation was calculated in order to obtain the temperature increase per second. The specific heat and density values of the suspensions were approximated to those of water and the heat needed to produce this temperature increase in each well was determined. The heating efficiency was calculated using these heat values and the laser power of 1,100 mW.

### *In vitro* studies

All *in vitro* studies were performed using Vero cell line (kidney epithelial cells from African green monkey), acquired from the American Type Culture Collection (ATCC: CCL-81). Cells were cultured at 37°C in a 5% CO_2_ atmosphere in Dulbecco's modified Eagle's medium (DMEM) supplemented with 10% fetal bovine serum (FBS), 2 mM glutamine, and 100 U/mL penicillin/streptomycin.

### MTT cell viability assays

Vero cells were seeded at a density of 5 × 10^3^ cells per well in a 96-multiwell plate and incubated with the nanomaterials for 24 h, as previously described. At this point, cells were irradiated with the NIR laser and incubated for 5 h under cell culture conditions. Thereafter, medium was removed and cells were incubated with 200 μL of DMEM containing 10 μL of 5 mg/mL MTT in the dark, under culture conditions, for 90 min. Finally, the plate was centrifuged at 1,250 g for 30 min using an Eppendorf centrifuge 5810R with an A-4-62 rotor, the supernatant was removed and the formazan crystals were solubilised with 100 μL of dimethyl sulfoxide (DMSO). After mixing, the optical density at 555 nm was recorded using a plate reader. Experiments were performed in quintuplet to determine the standard deviations. Experiments were reproduced in triplicate to verify the reproducibility of the results.

### Thermoablation conditions

Vero cells were seeded at a density of 5 × 10^3^ cells per well in a 96-multiwell plate and incubated under cell culture standard conditions. After 24 h the medium was replaced for fresh DMEM with the desired concentrations of each nanomaterial (ranging from 0 to 60 μg of gold/mL with a final water concentration of 10%) and incubated for another 24 h under culture standard conditions. Thereafter, the cells were washed twice with DPBS to remove any excess of remaining particles or dead cells and fresh phenol red-free DMEM was added. Samples were performed in triplicate and irradiated under working laser configuration for 10 min, with temperature control set at 37°C. Non-irradiated samples of cells with and without nanomaterials and irradiated cells (without nanomaterials) were used as controls. Representative images were collected 5 h post-irradiation.

### Live/dead cell viability assays

LIVE/DEAD™ Viability/Cytotoxicity Kit tests were performed according to the instructions of the supplier. Briefly, DMEM of irradiated samples and their respective controls was removed after 5 h under standard culture conditions. Thereafter, cells were incubated for 30 min at room temperature (protected from light) with 100 μL of DPBS containing calcein (2,000 times diluted from the stock solution) and Ethidium Bromide homodimer (500 times diluted from stock solution). Finally, bright field and fluorescent images of the cells were acquired using the microscope previously described employing 472 ± 30/520 ± 35 nm (GFP) and 531 ± 46/593 ± 40 nm (TRITC) cube filters. Images of all studied conditions were acquired employing identical optical parameters in the microscopy in order to be comparable.

## Results and discussion

### AuNR entrapment in a chitosan hydrogel and characterisation

The synthesis of high aspect ratio AuNRs was performed following our previously reported seed mediated growth process (Alfranca et al., 2016). AuNRs with a LSPR at 1,040 nm, close to the 1,064 nm wavelength of the laser used in the photothermal therapy and well within the NIR biological window were obtained. This is of great importance because at this wavelength the absorption of light by tissues and cells is highly decreased, meaning that heating will only be produced in the presence of AuNRs and so reducing damage to cells that are not interacting with AuNRs.

**Scheme 1:**
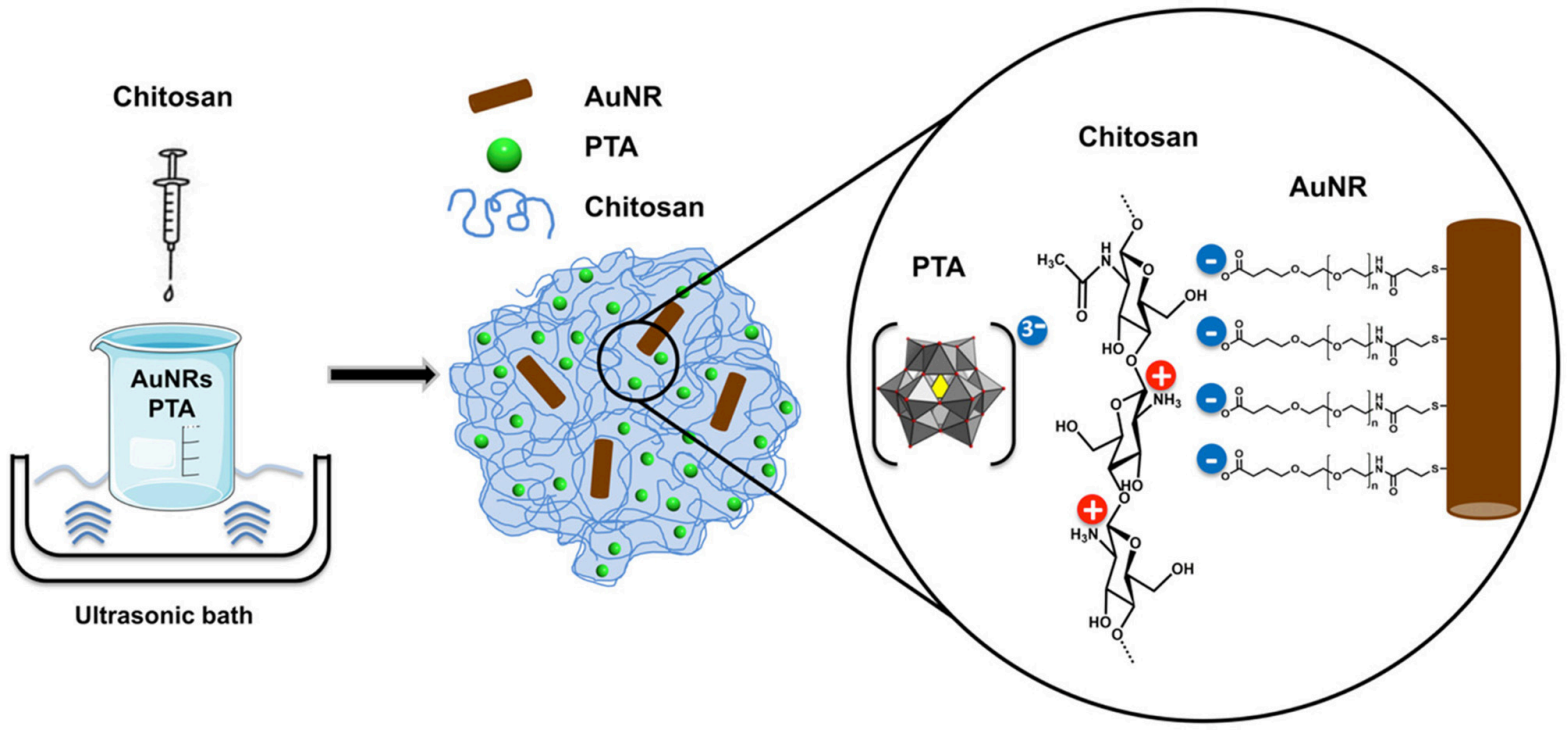
PTA-mediated synthesis of the AuNR@CS hydrogel nanocontainer.

The overall aim of entrapping this photothermal agent in the cell-adhesive chitosan was to enhance the AuNR interaction with cells. It has been proven that chitosan possesses excellent mucoadhesive properties enhancing the retention in mucosal tissues (Ways et al., 2018). To take advantage of this property, our hybrid material was synthesized by an ionotropic gelation method, in which the polymer matrix is formed by interaction of the positively charged amino groups in chitosan with a negatively charged gelling agent (See Scheme 1). In this case, the gelling agent of choice was the Keggin-type polyoxometalate (POM) phosphotungstic acid (PTA), which gives structural stability to the hydrogel matrix. PTA was chosen based on previous studies illustrating its ability to form spherical and biocompatible nanocapsules (De Matteis et al., 2014).

One of the key advantages of this synthesis method is that it is performed in aqueous solution, making it compatible with all the components involved in the hybrid hydrogel (CS, PTA, and AuNR). The synthesized hydrogel was fully characterized to investigate the chemical composition, structure, and morphology, as well as to confirm the presence of AuNRs in the hydrogel matrix and the suitability of the developed system for its application in photothermal therapy.

**Figure 1:**
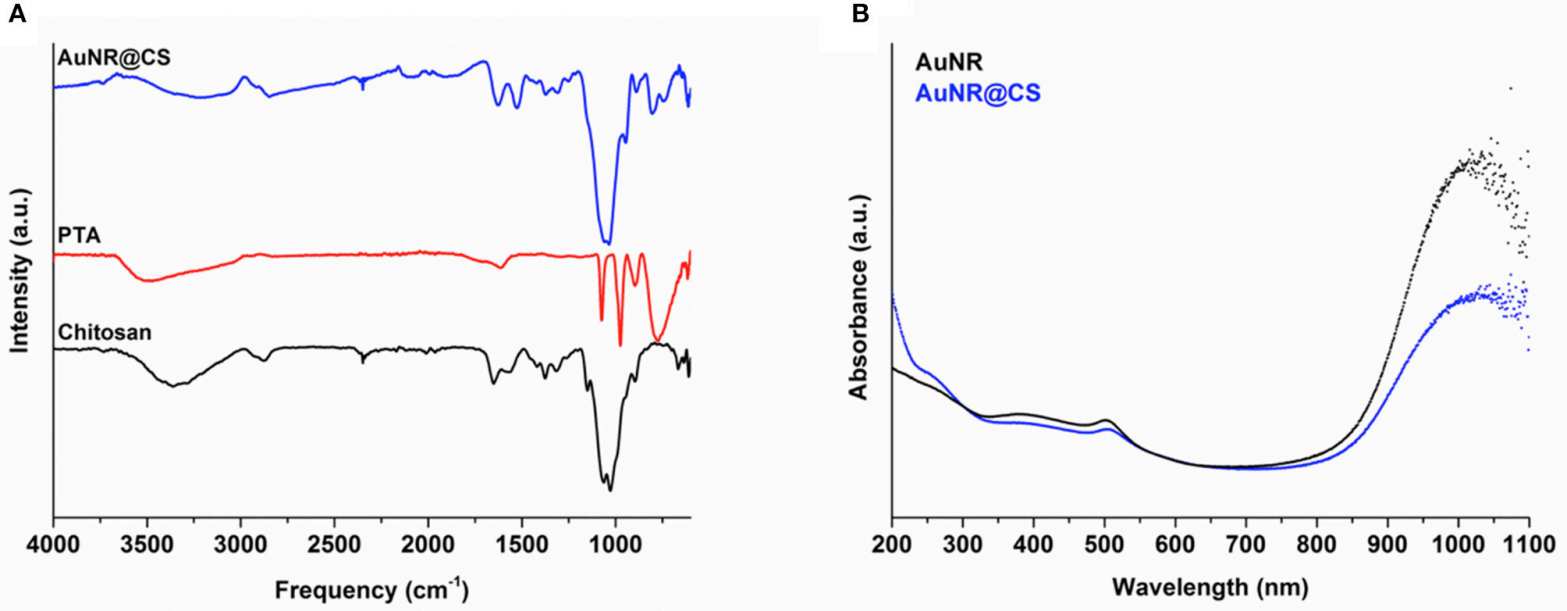
**(A)** FTIR spectra of AuNR@CS and free PTA and chitosan. **(B)** Aqueous UV-vis spectra comparing free AuNR with AuNR@CS.

FTIR spectroscopy was used to determine the composition of hybrid organic-inorganic matrix. FTIR data in Figure 1 show the most important vibrational peaks of chitosan: 1,630 (primary amide), 1,414 (–CH_2_), 1,375 (–CH_3_), and 1,150 cm^−1^ (C–O–C skeletal vibrations). Protonated amino group appears at 1,530 cm^−1^ only for the AuNR@CS, probably due to the fact that in the case of AuNR@CS amino groups are electrostatically interacting with negative charges of PTA and/or PEG in the surface of AuNRs. The presence of the POM is largely masked by the chitosan stretches, but can be confirmed by the appearance of small peaks in the AuNR@CS spectrum below 1,000 cm^−1^, corresponding to P-O and W = O stretches. AuNR peaks cannot be observed in the AuNR@CS, because their intensity is too low in comparison with other spectral bands.

The AuNRs possess a distinctive LSPR band at a wavelength of 1,040 nm, which is highly appropriate for PTT using a 1,064 nm NIR laser. Confirmation of the presence of AuNR in the polymer matrix was performed by UV-vis spectrophotometry. This technique shows that the characteristic LSPR peak at 1,040 nm of free AuNR is still present in AuNR@CS, supporting the fact that the plasmonic properties of AuNRs were maintained after their entrapment and that they were not affected by their close proximity within the hydrogel matrix. Both spectra also show an absorbance peak at 505 nm, corresponding to transversal absorbance band of AuNRs.

The amount of gold from AuNR entrapped in the hydrogel was precisely quantified using ICP-AES. Correlating the Au concentration obtained from ICP-AES analysis of the hybrid matrix with the initial amount of Au added in the synthesis, we obtained an encapsulation efficiency (mg of encapsulated AuNR/mg of AuNR initially added × 100) of 61%. Moreover, an AuNR loading (mg of encapsulated AuNR/mg of hydrogel × 100) of 28 % was obtained in the final material.

**Figure 2:**
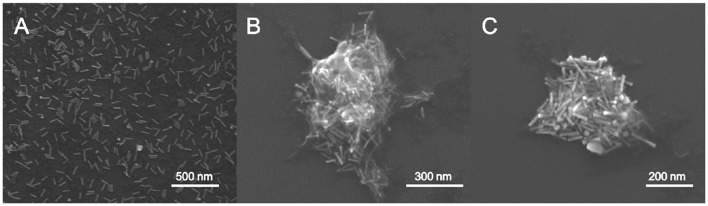
Scanning electron microscopy (SEM) image of AuNR **(A)** and AuNR@CS **(B,C)**.

SEM was used to study the morphology of the hybrid matrix and to confirm the presence of the AuNRs inside the chitosan hydrogel (Figure 2). SEM images of the AuNR@CS nanocontainers were obtained by fixing the hydrogel by glutaraldehyde covalent crosslinking. Glutaraldehyde acts as a crosslinker between the amino groups in chitosan polymer chains, rigidifying the hydrogel structure and helping to maintain it under electron microscopy conditions. Using this method sub-micron containers ranging from 200 to 500 nm in diameter were observed, all of them containing AuNRs embedded in their structure (Figures 2B,C). From Figures 2B,C it is possible to observe how the polymeric structure was affected by the microscope conditions (high vacuum) even after glutaraldehyde crosslinking. However, in both images polymer residues are clearly observed surrounding the AuNRs. This sub-micrometric size of the hydrogel structure was confirmed by DLS analysis by means of photocorrelation spectroscopy in which a mean hydrodynamic diameter of 475 nm was obtained. The electrophoretic mobility of the container was also evaluated. In this case, a Zeta Potential of +24.4 mV was obtained, which is in agreement with the composition of the hydrogel, whereby protonated amino groups in the chitosan polymer structure confer a positive potential to the material.

### Cytotoxicity of AuNR@CS

The cytotoxicity of the hydrogel nanocontainers was assessed prior to further testing and analysis. Increasing concentrations of sterile nanocontainers, from 0, 10, 50, 100 to 200 μg of capsules/mL (measured by dry weight), were incubated with Vero cells for 24 h, washed and the viability of the cells was evaluated by MTT cell viability assay (Supplementary Figure 2). In this assay, NAD(P)H-dependent cellular oxidoreductase enzymes reduce MTT reagent to its purple colored product showing both differences in the number of remained viable cells and their metabolic activity. The lack of any cytotoxicity from AuNR@CS at the highest tested concentration of 200 μg of capsules/mL serves as a first indication of their safety as photothermal agents for future *in vivo* studies.

### Heating profiles

**Figure 3:**
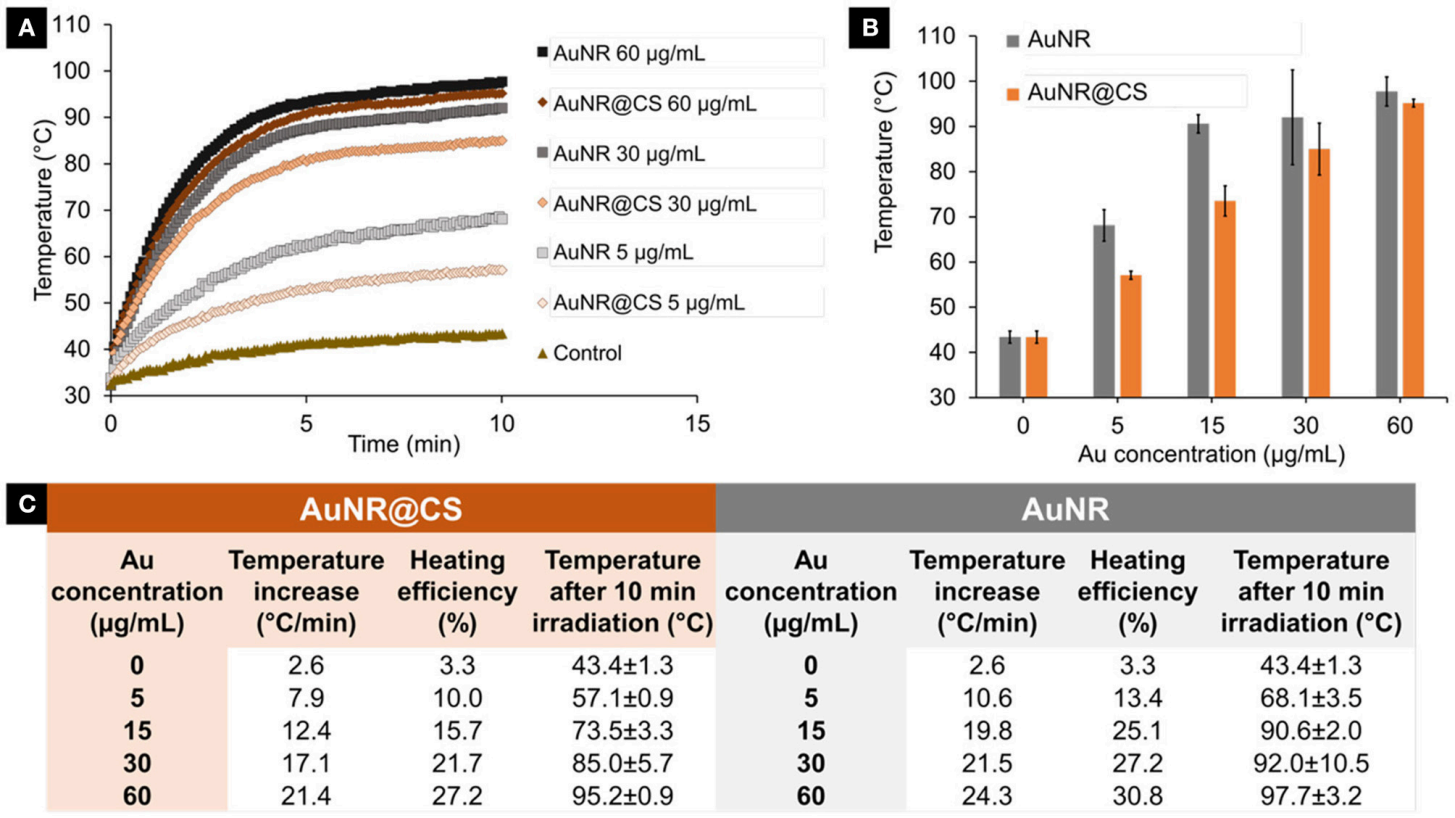
**(A)** Temperature increase during 10 min irradiation of different concentrations (5–60 μg/ml) of AuNR and AuNR@CS. **(B)** Final temperature after 10 min irradiation of different concentrations of AuNR and AuNR@CS. **(C)** General data table of all tested samples, including concentrations, initial temperature increase, heating efficiency, and final temperature after 10 min irradiation.

In order to evaluate if the entrapment of AuNR as part of the chitosan hydrogel affected their capability to convert light into heat, identical Au concentrations of AuNR and AuNR@CS diluted in Milli-Q water were compared. Their heating capability was investigated over a 10 min period of irradiation using a 1,064 nm NIR laser operating at 1,100 mW. Compared with the free AuNR, there was only a minor reduction in the initial temperature increase of AuNR@CS (3–7°C/min) as can be seen in Figure 3. We attribute this reduction in temperature increase to a combination of thermal insulation caused by the hydrogel and weak interactions between the LSPR bands of other nearby AuNRs. In any case, the final global temperatures following 10 min of irradiation were highly increased both in the case of AuNR and AuNR@CS at all concentrations studied (see Figure 3B), compared to water control that only reached 43.4 ± 1.3°C. These results suggested that irradiation at tested conditions should be harmless for cells and biological tissues without nanoparticles and they also demonstrated the suitability of these nanomaterials for photothermal treatment.

The heating power and the heating efficiency (see Materials and Methods section) for each of the tested concentrations were quantified by calculating the initial heating increase per second (See SI, Supplementary Figure 1). As a result, the heating efficiency of AuNRs ranged from 13 to 31% depending on the concentration studied and from 10 to 27% in the case of AuNR@CS as described in the Figure 3.

### Improving *in vitro* photothermal therapy of AuNR via chitosan entrapment

**Figure 4:**
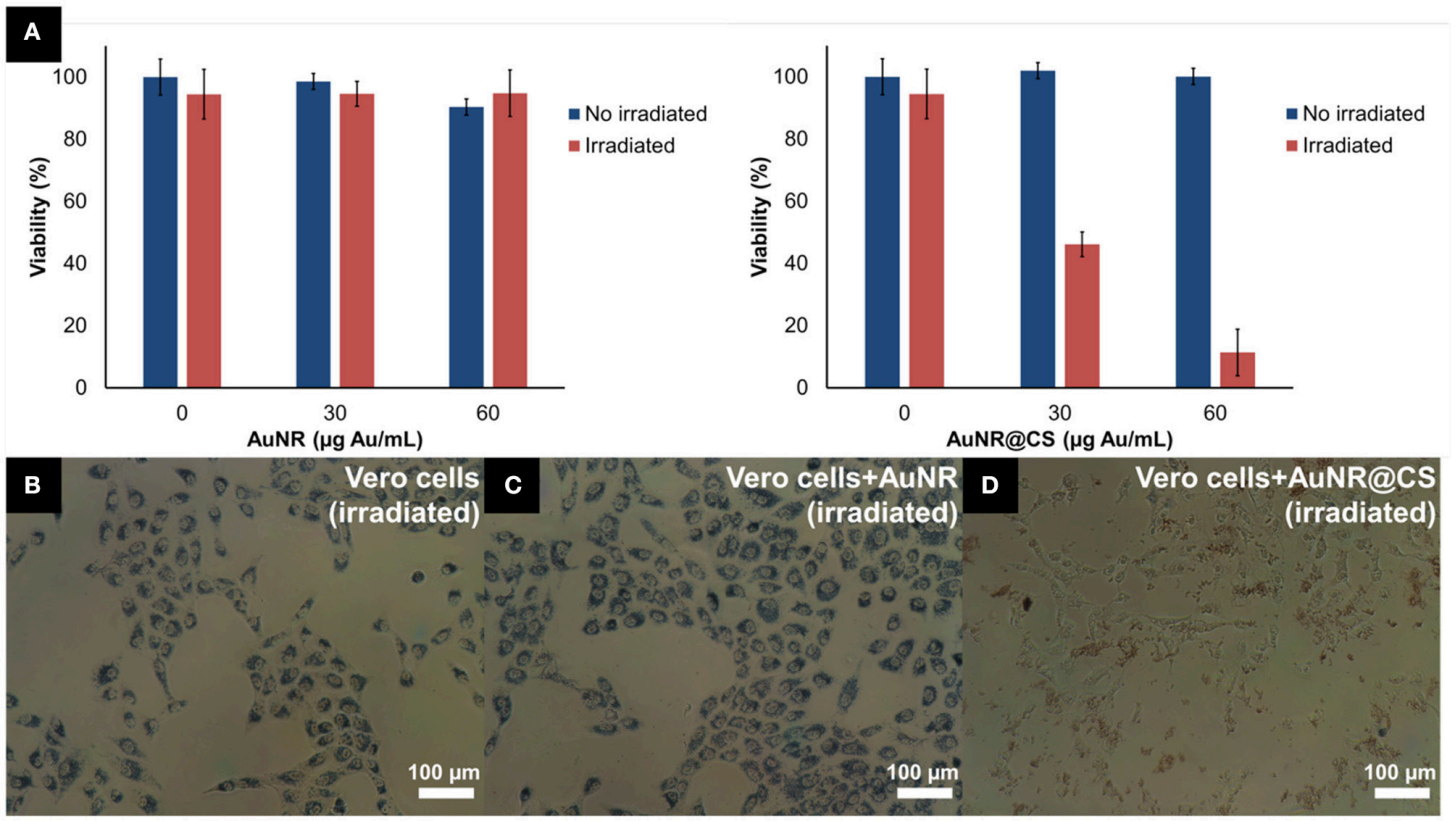
**(A)** MTT cell viability of Vero cells incubated with different concentrations of AuNR or AuNR@CS 5 h after laser irradiation. **(B–D)** Phase contrast microscopy images of cells during MTT incubation, after irradiation: untreated cells **(B)**, 60 μg/mL of AuNR **(C)**, or 60 μg/mL of AuNR@CS **(D)**.

In previous work we have shown how AuNRs possessed a high potential for photothermal applications due their efficacy as light-to-heat transducers; however, this potential was affected by the fact that AuNR suffer from very poor cellular internalization when compared with other gold nanoparticles (Alfranca et al., 2016). The entrapment of AuNRs in a cationic chitosan hydrogel matrix using an anionic POM as gelling agent allowed us to overcome this limitation for the use of AuNRs in photothermal applications—while simultaneously avoiding the use of complex AuNR surface functionalization strategies. Optical microscopy images taken during the MTT assays illustrated how the AuNR@CS showed a cell-adhesive behavior and confirmed that the AuNR@CS were still interacting with the surface of the cells even after several washing steps, as can be seen in Figure 4D.

The low cytotoxicity combined with cell-adhesive nature of the AuNR@CS make them promising candidates for improving photothermal therapy. The excess of material that was not interacting with cells was removed with PBS before irradiating the cells for 10 min using a 1,064 nm NIR laser operating at 1,100 mW. This allowed a very localized delivery of heat in cells that contained AuNR, or in which AuNRs were interacting with their surfaces. Five hours after irradiation, morphological changes could be clearly seen in the case of cells incubated with AuNR@CS (Supplementary Figure 3); while no morphological changes were observed in the case of irradiated control cells and irradiated cells incubated with AuNR.

MTT cell viability assay was used to quantify the photothermal behavior of AuNR@CS 5 h after irradiation. This study was carried out 5 h after irradiation because at this time the effects of apoptosis or secondary necrosis were observed in previous PTT studies (Pérez-Hernández et al., 2015). The results of these experiments confirmed that there was no associated toxicity of both AuNR and AuNR@CS in non-irradiated cells. The absence of cell death derived from the laser and the AuNR was demonstrated using laser-irradiated control cells (without nanorods) and cells incubated with AuNR, respectively. In contrast, for irradiated cells treated with AuNR@CS the cell viability showed a marked decrease to 46.1 ± 7.5% at a concentration of Au entrapped in the hydrogel of 30 μg/mL and a more dramatic decrease (11.4 ± 1.7% of unaffected cells) at 60 μg/mL of entrapped Au (corresponding to a dry weight of matrices of 200 μg/mL; Figure 4A). It should be noted that irradiated cells incubated with AuNR@CS at 60 μg/mL of gold failed to reduce the MTT reagent, indicating severe cellular damage, as observed in Figure 4.

**Figure 5:**
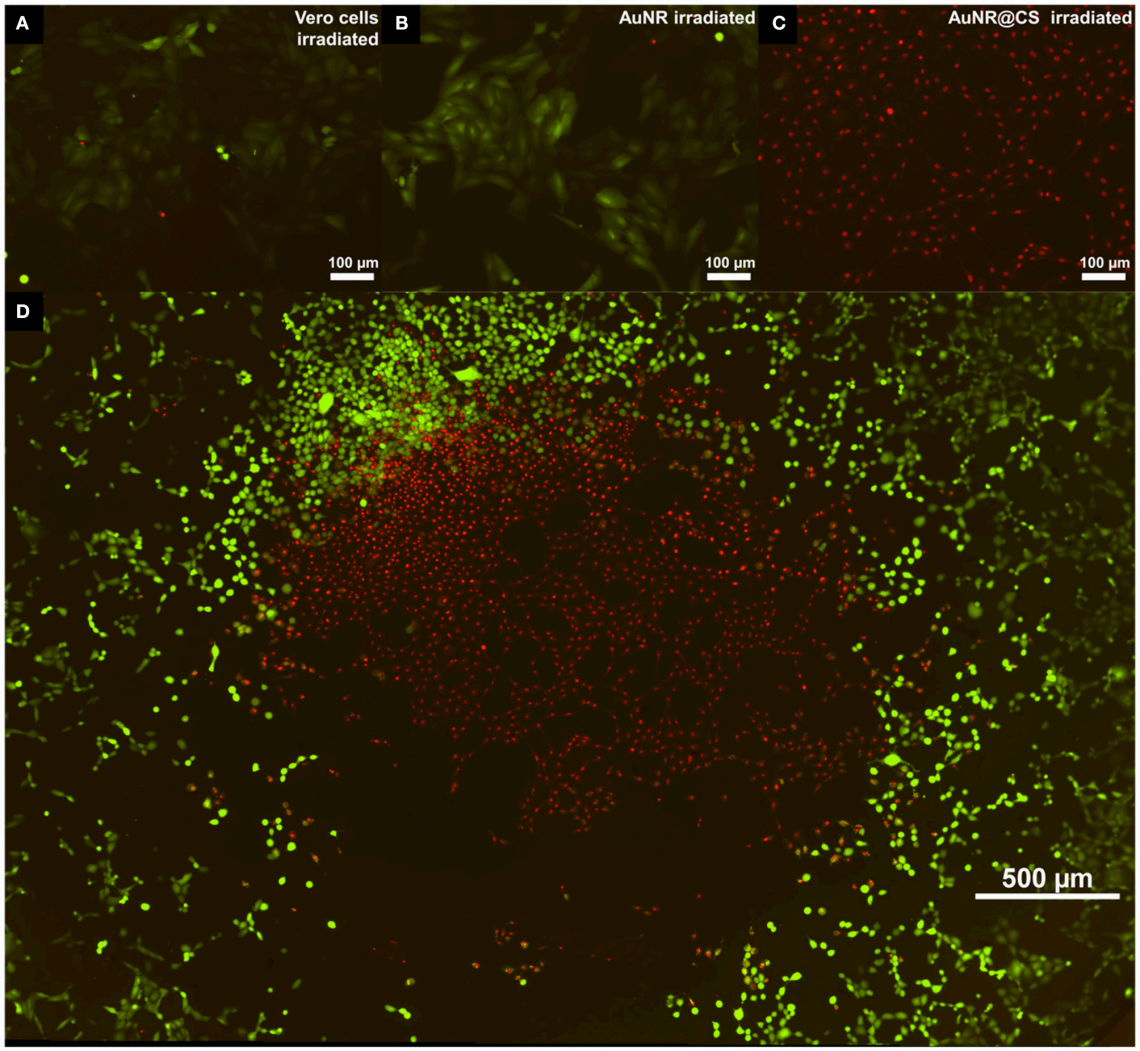
Fluorescent microscopy images of live/dead test 5 h post irradiation of untreated cells **(A)**; treated with 60 μg/mL of AuNR **(B)**; or with 60 μg/mL of AuNR@CS **(C)**. Image **(D)** shows an entire 96-well irradiation of 60 μg/mL AuNR@CS with the laser adjusted to irradiate only the central part of the well.

To confirm the results and observations from the MTT assay and to study how irradiation of the cells in the presence of the nanomaterials affects the integrity of cell membranes, a commercial LIVE/DEAD™ test was carried out 5 h after irradiation (See SI, Supplementary Figure 4). In this test, the intracellular esterase activity of live cells transform calcein AM to the green fluorescent calcein; while ethidium homodimer only enters into dead cells with damaged plasma membranes, increasing its red fluorescence when it interacts with nucleic acids. Irradiated control cells and cells incubated with AuNR displayed only live cells (Figures 5A,B, respectively); however, in the case of cells incubated with AuNR@CS the majority of irradiated cells were red stained, indicating cell death (Figure 5C). In addition, when the laser was adjusted to irradiate only the central part of the well of cells incubated with AuNR@CS, hyperthermia affected only cells in the irradiated area, while the remaining cells in the well remained alive (Figure 5D). These results confirmed that by entrapping the AuNRs in the cell-adhesive chitosan hydrogel we could dramatically increase their efficiency as photothermal agents *in vitro*, thereby turning ineffective free AuNRs into highly applicable nanomaterials. These promising *in vitro* results provide the first lines of evidence that the AuNR@CS nanocontainers present potential application for the treatment of certain tumors. Further, the hybrid composite would be of particular use in intratumoural administration or after surgical removal of tumors, similar to other recent applications of hybrid gold nanoparticle materials (Conde et al., 2016). In addition, these AuNR@CS could be employed for gastrointestinal tumor treatment, as other chitosan hybrid nanomaterials have demonstrated to be able to effectively cross the intestinal epithelia and displayed tumor reduction with longer survival rates *in vivo* (Kang et al., 2017).

## Conclusions

This work describes the development of a simple strategy to significantly increase the efficacy of AuNRs for photothermal therapy applications *in vitro*. Although AuNRs are important class of photothermal nanoparticles, their cellular internalization is limited and must be increased using complex and time-consuming surface-functionalization techniques. As an alternative strategy, we have entrapped AuNRs in chitosan-based sub-micrometric hydrogels (AuNR@CS) that showed cell-adhesive properties. Importantly, an anionic polyoxometalate (POM) was employed as a gelling agent to control the sub-micrometric size of the hydrogel matrix. In addition, the anionic POM completes the ionic interaction with cationic chitosan to capture the AuNRs as part of the hydrogel. The AuNR@CS showed no cytotoxicity, even at high concentrations; however, laser irradiation of cells treated with AuNR@CS displayed clear signs of necrosis. In contrast, cells treated with free AuNR were completely unaffected, showing almost 100% viability. Consequently, our hydrogel entrapment strategy has increased the *in vitro* efficiency of AuNRs for photothermal therapy applications and could potentially be employed in intratumoural administration, resection of tumors zones after surgery or for oral delivery to treat tumors located in the digestive tract.

## Author contributions

ÁA and SG-E performed all the experimental research under the supervision and guidance of LD, SM, and JdlF. All authors were involved in writing the manuscript.

Heating profiles of laser irradiated nanomaterials, MTT cell viability assays, phase contrast microscopy, and fluorescence microscopy images of cells treated with AuNRs@CS.

### Conflict of interest statement

The authors declare that the research was conducted in the absence of any commercial or financial relationships that could be construed as a potential conflict of interest. The handling Editor and reviewer TP declared their involvement as co-editors in the Research Topic, and confirm the absence of any other collaboration.
