# Supplementary material for: Effective in Vitro Photokilling by Cell-Adhesive Gold Nanorods
Source: Front Chem. 2018 Jun 22;6:234. doi: 10.3389/fchem.2018.00234 (PMC6024193; doi:10.3389/fchem.2018.00234)
Supplement: Supplementary file 1 [file Data_Sheet_1.DOCX]

Supplementary Material

Effective in vitro photokilling by cell-adhesive gold nanorods

Álvaro Artiga^1‡^, Sonia García-Embid^1‡^, Laura De Matteis^2*^ and Scott G. Mitchell1^*^, Jesús M. de la Fuente^1^

1. Instituto de Ciencia de Materiales de Aragón (ICMA), Consejo Superior de Investigaciones Científicas (CSIC)-Universidad de Zaragoza

2. Instituto de Nanociencia de Aragón (INA), Universidad de Zaragoza, Spain

^‡^ Authors contributed equally

*** Correspondence:** Laura De Matteis [lauradem@unizar.es](mailto:lauradem@unizar.es) and Scott G. Mitchell [scott@unizar.es](mailto:scott@unizar.es)

Keywords: polyoxometalate, gold nanorod, chitosan hydrogel, encapsulation, photothermal therapy, near infra-red.


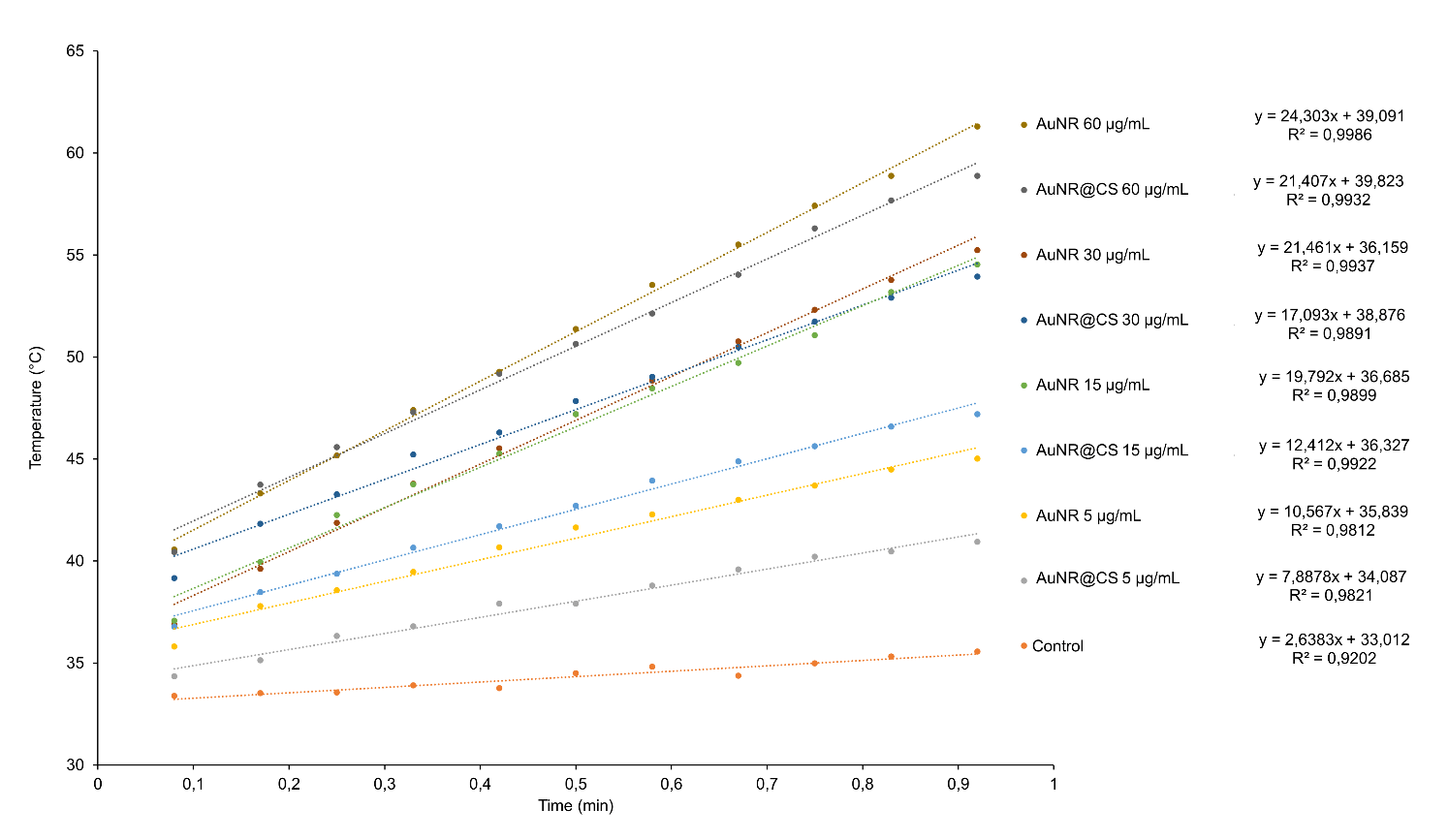


**Supplementary Figure 1**. Linear regression of the temperature increase *vs.* the first minute of irradiation for different concentrations of AuNR and AuNR@CS. The slopes of these regressions correspond to the initial temperature increase and was employed to calculate the heating efficiency of each sample.


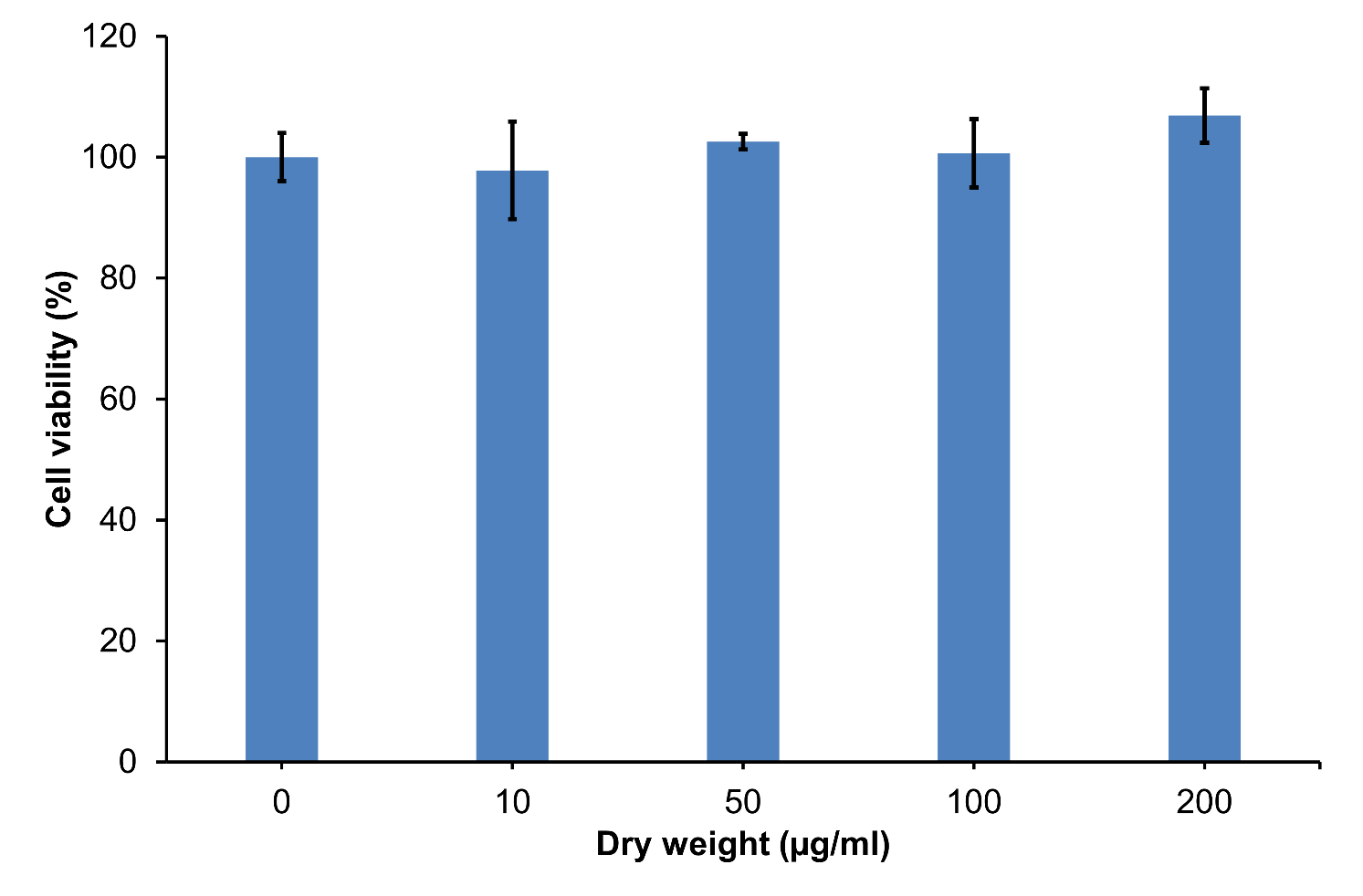


Supplementary Figure 2. MTT cell viability assay of Vero cells incubated with different concentrations of AuNR@CS showing no cytotoxicity even at highest concentration of 200 μg/mL.


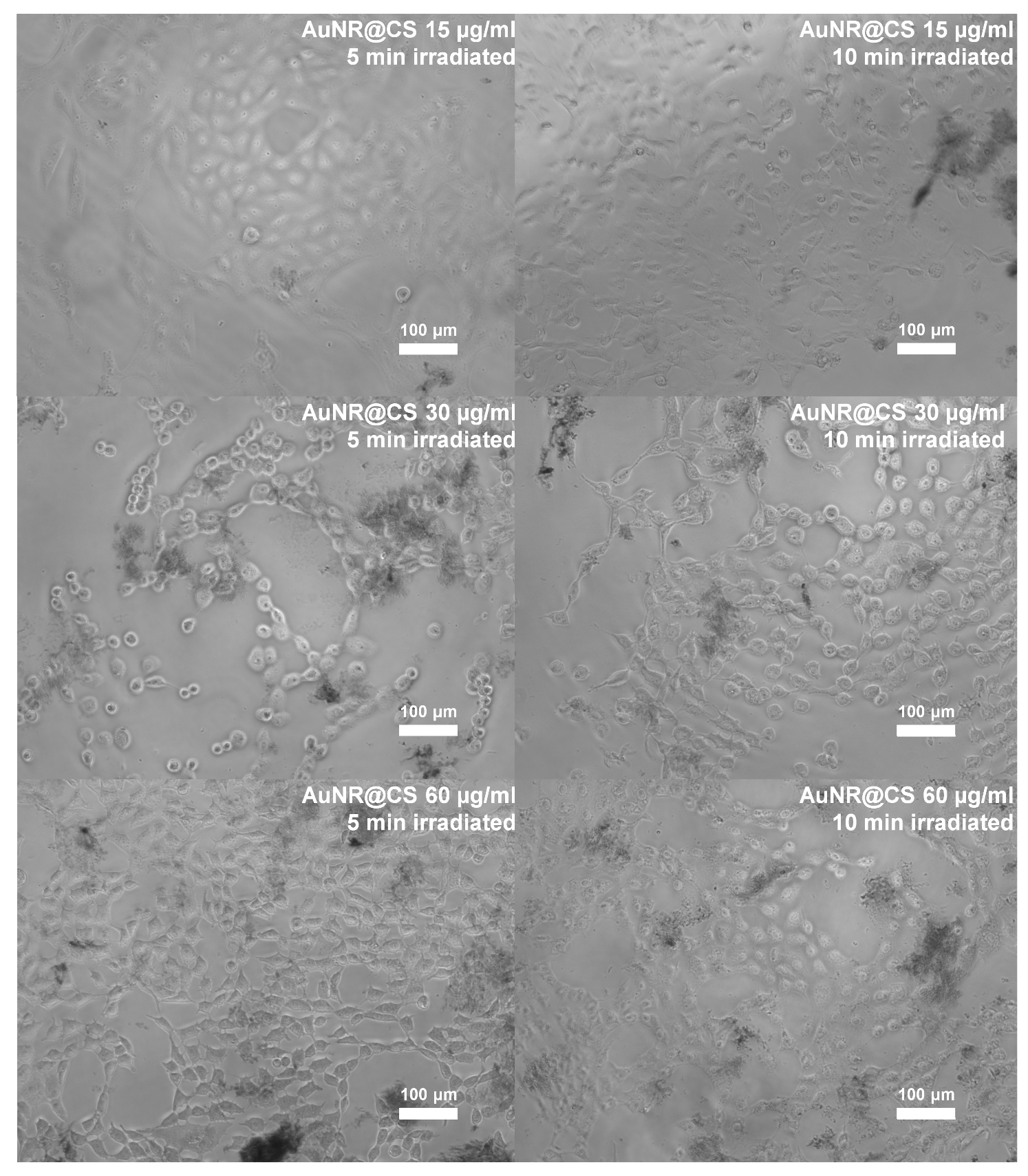


**Supplementary Figure 3**. Phase contrast microscopy images taken after 5 hours post-irradiation showing the morphology of Vero cells treated with different concentrations of AuNR@CS irradiation for 5 or 10 minutes, respectively.

**
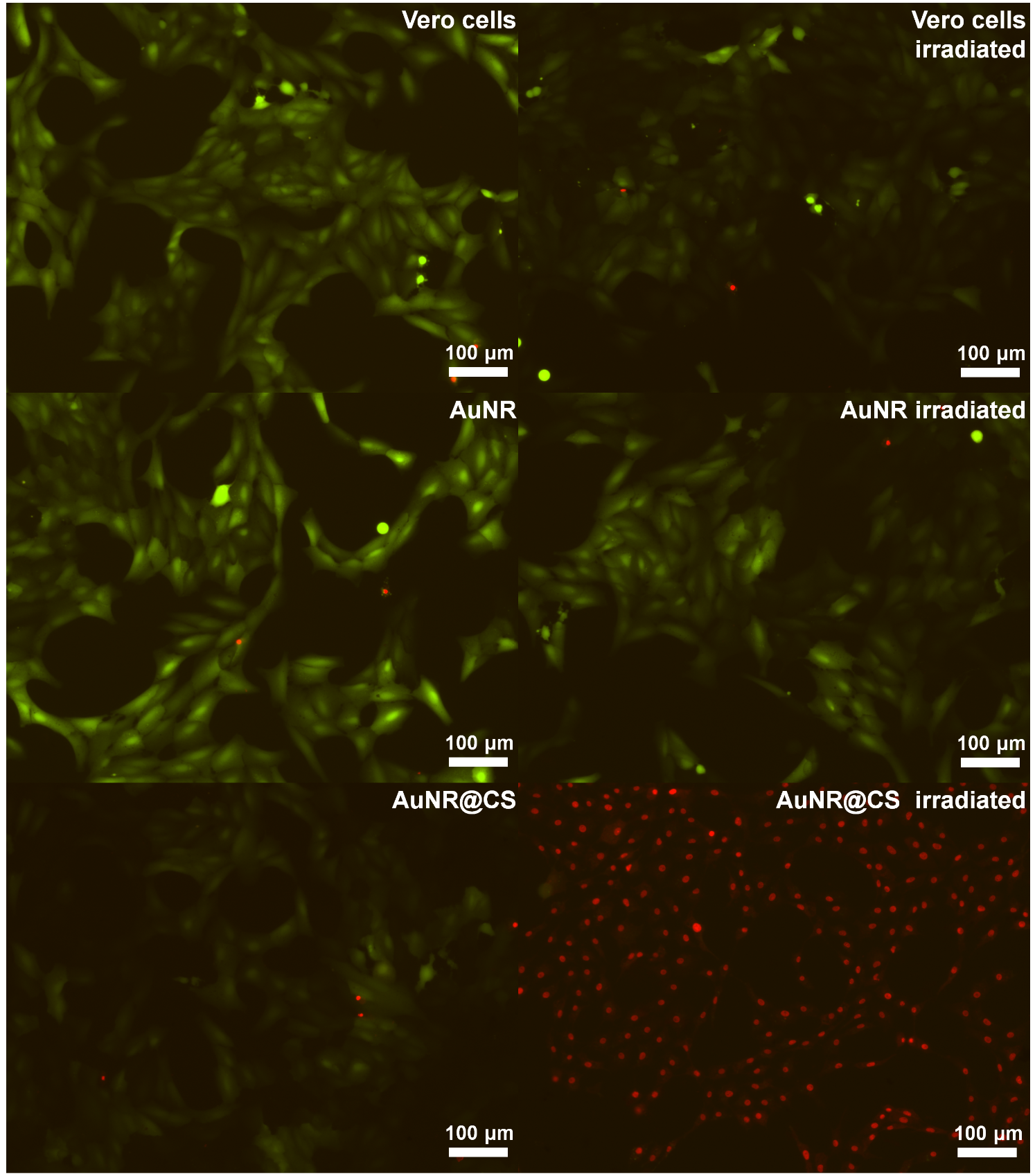
**

**Supplementary Figure 4**. Fluorescent microscopy images of Live/Dead test showing cells treated with 60 µg/mL of AuNR or AuNR@CS along with the untreated (control) sample. All images were taken 5 hours post-irradiation (non-laser irradiated samples were taken at the same time, without laser irradiation at time 0).
